# Clinical outcomes and prognostic factors of patients with sepsis caused by intra-abdominal infection in the intensive care unit: a post-hoc analysis of a prospective cohort study in Korea

**DOI:** 10.1186/s12879-022-07837-x

**Published:** 2022-12-19

**Authors:** Chan Hee Park, Jeong Woo Lee, Hak Jae Lee, Dong Kyu Oh, Mi Hyeon Park, Chae-Man Lim, Suk-Kyung Hong, the Korean Sepsis Alliance (KSA) investigators, Chae-Man Lim, Sang-Bum Hong, Dong Kyu Oh, Gee Young Suh, Kyeongman Jeon, Ryoung-Eun Ko, Young-Jae Cho, Yeon Joo Lee, Sung Yoon Lim, Sunghoon Park, Chae-Man Lim, Suk-Kyung Hong, Sang Hyun Kwak, Song-I. Lee, Jae Young Moon, Kyung Chan Kim, Sunghoon Park, Tai Sun Park, Youjin Chang, Gil Myeong Seong, Heung Bum Lee, Jeongwon Heo, Jae-myeong Lee, Woo Hyun Cho, Kyeongman Jeon, Yeon Joo Lee, Sang-Min Lee, Su Hwan Lee, Jong-Joon Ahn, Eun Young Choi

**Affiliations:** 1 Division of Trauma Surgery, Department of Surgery, Dongsan Medical Center, Keimyung University School of Medicine, Daegu, Republic of Korea; 2 Division of Acute Care Surgery, Department of Surgery, Asan Medical Center, University of Ulsan College of Medicine, 88 Olympic-Ro 43-Gil, Songpa-Gu, Seoul, 05505 Republic of Korea; 3 Department of Pulmonary and Critical Care Medicine, Asan Medical Center, University of Ulsan College of Medicine, Seoul, Republic of Korea

**Keywords:** Intensive care unit, Intra-abdominal infection, Mortality rate, Organ dysfunction, Sepsis, Source control

## Abstract

**Background:**

Sepsis is the most common cause of death in hospitals, and intra-abdominal infection (IAI) accounts for a large portion of the causes of sepsis. We investigated the clinical outcomes and factors influencing mortality of patients with sepsis due to IAI.

**Methods:**

This post-hoc analysis of a prospective cohort study included 2126 patients with sepsis who visited 16 tertiary care hospitals in Korea (September 2019–February 2020). The analysis included 219 patients aged > 19 years who were admitted to intensive care units owing to sepsis caused by IAI.

**Results:**

The incidence of septic shock was 47% and was significantly higher in the non-survivor group (58.7% vs 42.3%, *p* = 0.028). The overall 28-day mortality was 28.8%. In multivariable logistic regression, after adjusting for age, sex, Charlson Comorbidity Index, and lactic acid, only coagulation dysfunction (odds ratio: 2.78 [1.47–5.23], *p* = 0.001) was independently associated, and after adjusting for each risk factor, only simplified acute physiology score III (SAPS 3) (*p* < 0.001) and continuous renal replacement therapy (CRRT) (*p* < 0.001) were independently associated with higher 28-day mortality.

**Conclusions:**

The SAPS 3 score and acute kidney injury with CRRT were independently associated with increased 28-day mortality. Additional support may be needed in patients with coagulopathy than in those with other organ dysfunctions due to IAI because patients with coagulopathy had worse prognosis.

## Introduction

Sepsis is an uncontrolled reaction of the host to infection, which can be potentially life-threatening. Accurate calculation of the global burden of sepsis is difficult. A recent study has reported approximately 48.9 million cases and 11 million sepsis-related deaths in 2017. This accounts for nearly 20% of all deaths worldwide, and sepsis is the most common cause of death in hospitals in the United States [1, 2]. Sepsis is a major public health issue, and the costs for treating sepsis in hospitals have increased, exceeding US $24 billion per year [2, 3]. Likewise, the incidence of sepsis and the treatment costs in Korea have steadily increased, particularly among older adults [4].

Intra-abdominal infection (IAI) is defined as an inflammatory reaction to bacteria and their toxins in the peritoneum, resulting in a purulent exudate in the peritoneal cavity [5–8]. Among all the causes of sepsis, IAI is reportedly the second most common cause, with a relatively high mortality rate of nearly 30.0% [4, 9, 10]. In particular, most sepsis cases in surgical intensive care units (ICUs) are caused by IAI. Source control and antibiotic use are essential in the treatment of these patients. Various forms of IAIs may exist, including those in the hepatobiliary tract, stomach, small bowel, and colon. In addition, there are differences in mortality and clinical outcomes according to the type of organ, degree of anatomical disruption, and duration of IAI [5, 7, 9]. Treatment of IAI can be difficult because the spectrum of infection is wide compared with that of other infection causes and source control, wherein drainage or surgical treatment is often required.

However, studies on the clinical outcomes and impact of organ dysfunction in patients with sepsis due to IAI are limited. Therefore, the primary objective of our study was to investigate the clinical outcomes and the factors affecting the mortality in patients with sepsis due to IAI. The secondary objective was to determine the impact of organ dysfunction on mortality rates.

## Materials and methods

### Study design, setting, and definition

This post-hoc analysis of a prospective cohort study was performed by the Korean Sepsis Alliance (KSA) encompassing 16 tertiary or university-affiliated hospitals in Korea. The Steering Committee developed the research data collection methods to manage the sepsis data platform, periodically reviewed the progress of each study, and supervised the overall research progress in association with the Korea Disease Control and Prevention Agency (KDCA). The data used in this study were screened from all consecutive patients who visited the participating hospitals for six months (September 1, 2019, to February 29, 2020).

This prospective cohort study analyzed data from the Korean sepsis registry, and this study was already deliberated as a service project by the KDCA. The study was approved by the institutional review boards (IRB) of all participating hospitals, including the IRB of Asan Medical Center (approval number 2018-0675). Data were collected and analyzed in an ethical manner while protecting the patients' right to privacy. The requirement for informed consent was waived owing to the non-interventional, observational characteristics by the IRB of all participating hospitals, including the IRB of Asan Medical Center (approval number 2018-0675).

**Fig. 1:**
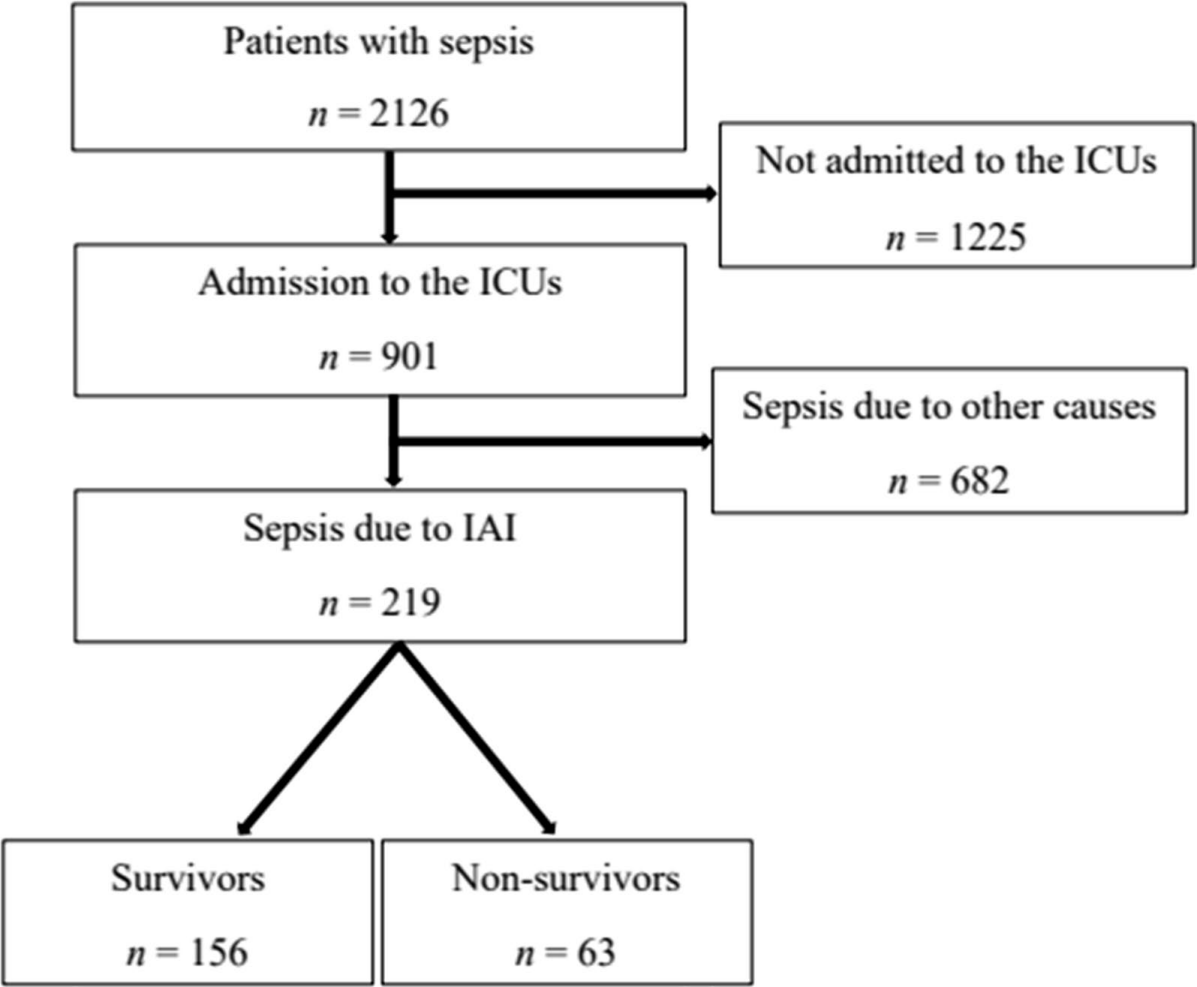
Flow chart of patients’ enrollment. *ICU* intensive care unit, *IAI* intra-abdominal infection

Our study enrolled a total of 2126 patients with sepsis who were admitted to the participating hospitals for 6 months. Of these patients, 901 were treated in ICUs. Finally, the study included 219 patients who were admitted to the ICU due to sepsis caused by IAI (Fig. 1). Sepsis patients aged ≥ 19 years were included and followed up until death or discharge. As defined by the Clinical Criteria of the Third International Consensus Definition (Sepsis-3), sepsis was defined as a life-threatening organ dysfunction resulting from an uncontrolled host response to infection [11]. Organ dysfunction was included in the definition of sepsis, and the presence or absence of organ dysfunction was determined using a Sequential Organ Failure Assessment (SOFA) score. In this study, sepsis was diagnosed if the patient met the following two conditions: (1) suspicion or confirmation of infection and (2) increase in SOFA score by two points or more when an event occurred. The base SOFA score of each organ was assumed to be zero for patients with no known pre-existing organ dysfunction, and patients with a SOFA score of two or higher at the time of the event were enrolled in the study. Peritonitis caused by perforation of a hollow viscus, intra-abdominal abscess, biliary tract infection, pancreatic infection, enteritis, and colitis were defined as having IAI. The Eastern Cooperative Oncology Group (ECOG) scale is a measurement index created to check performance status, determine appropriate treatment policy, and predict prognosis in cancer patients. Using the ECOG scale, it is possible to objectively evaluate the patient's level of functioning in terms of their ability to care for themselves, perform activities of daily living, and their physical ability. It is divided into six scales: (0) fully active and no performance restrictions; (1) strenuous physical activity restricted, fully ambulatory and able to carry out light work; (2) capable of all self-care but unable to carry out any work activities; up and about > 50% of waking hours; (3) capable of only limited self-care and confined to bed or chair > 50% of waking hours; (4) completely disabled and cannot carry out any self-care; and (5) death. Continuous renal replacement therapy (CRRT) refers to patients who underwent CRRT for the treatment of acute kidney injury, and excludes patients who underwent conventional hemodialysis as renal replacement therapy. In community-acquired sepsis, time zero was defined as the time of an emergency room triage visit. In-hospital-acquired sepsis, time zero was defined as the first time the rapid response team recognized the sepsis. Source control was defined as non-surgical treatments such as percutaneous drainage, and surgical treatment such as debridement or laparotomy. Patients who were treated with antibiotics and received intensive care, without any such procedures, were considered not to have received source control.

### Data collection

All trained research coordinators in each participating hospital completed their entry into the shared data platform, and the coordinating hospital evaluated the quality of the data for completeness and logical errors. The data collected retrospectively in each hospital were as follows: (1) patient characteristics, including age, sex, body mass index (BMI), Charlson Comorbidity Index (CCI), physiological status, medical history, SOFA score, simplified acute physiology score III (SAPS 3), and laboratory data at time zero; (2) clinical results, including the duration from time zero to antibiotic administration, source control implementation, and duration from time zero to source control implementation; (3) infection and microbiological data, including the type of isolated bacteria and fungi, occurrence of bacteremia and multidrug resistance (MDR), and location of infection; and (4) organ dysfunction data, including the type of organ dysfunction results and analysis of the number of organ dysfunctions. Additional data compared with those in previous studies were collected. However, these were not used in our study.

### Statistical analysis

Statistical analysis was conducted by the staff of the coordinating center and by a professor at the Department of Clinical Epidemiology and Biostatistics, Asan Medical Center, who did not participate in data collection.

Data are presented as numbers (percentages) or as mean ± standard deviation. The characteristics and clinical data of the survivor and non-survivor groups were compared using the chi-square test or Fisher’s exact test (for categorical variables) and Student’s t-test (for continuous variables). Risk factors associated with 28-day mortality were analyzed by univariate and then multivariate logistic regression, and the degree of association with 28-day mortality was presented as an independent factor by means of odds ratios (ORs) with their corresponding 95% confidence intervals. Infection profile data were analyzed using the number (percentage) or mean ± standard deviation. The microbiological data were described by total numbers and proportions, which showed the distribution of bacteria and fungi in patients with sepsis due to IAI. Multivariate logistic regression analysis was used to estimate the association between organ dysfunction and 28-day mortality, with unadjusted and adjusted evaluations. We adjusted 28-day mortality for age, sex, CCI, and lactic acid levels. We used OR to present the impact of organ dysfunction on 28-day mortality and analyzed the impact of the number of organ dysfunctions on 28-day mortality using ORs in the logistic regression analysis. Also, the multiple logistic regression by stepwise selection was used to explain the 28-day mortality using independent variables. The logistic regression performance is denoted by c-statistics and Hosmer–Lemeshow tests. Differences were considered statistically significant at P < 0.05. All analyses were conducted using SPSS software (version 25.0; IBM Corp., Armonk, NY, USA).

## Results

### Patient characteristics and clinical data

During the 6-month study period, total 2126 patients were admitted owing to sepsis at the participating hospitals; 219 (10.3%) were admitted to the ICU owing to sepsis caused by IAI according to collected data review. The 28-day mortality rate of all enrolled patients with sepsis was 28.1% (n = 598/2126). Among these patients, the mortality rate was 28.8% (n = 63/219) for sepsis due to IAI in the ICU.

Patient characteristics and clinical data at the time of sepsis diagnosis are summarized in Table 1. The mean age of the patients was 69.4 ± 13.1 years, and 53.9% were men. The mean BMI was 23.0 ± 4.2 kg/m^2^; no significant difference was noted in BMI between the survivor and non-survivor groups. The mean CCI in the non-survivor group was higher than that of the survivor group, with significant differences (5.9 ± 3.0 vs. 5.1 ± 2.3, *p* = 0.011). The initial Eastern Cooperative Oncology Group scores ranged from 0 to 4. However, there was no significant difference in the scores between the two groups (*p* = 0.075). The incidence of septic shock was 47%, and was significantly higher in the non-survivor group than in the survivor group (58.7% vs. 42.3%, *p* = 0.028). The initial lactate level was significantly higher in the non-survivor group than in the survivor group (7.2 ± 5.0 vs. 4.5 ± 3.1, p < 0.001). In addition, the initial SOFA score was significantly higher in the non-survivor group than in the survivor group (12.2 ± 4.6 vs. 8.4 ± 3.1, p < 0.001). Moreover, patients who underwent CRRT had a higher mortality rate than those who did not undergo CRRT (68.3% vs. 16%, p < 0.001). By contrast, no significant difference was noted in the number of patients who were prescribed steroids to treat sepsis between the two groups (26.9% vs. 34.9%, *p* = 0.239). In the initial laboratory tests at time zero, total bilirubin, C-reactive protein, procalcitonin, and brain natriuretic peptide levels were higher in the non-survivor group.

**Table 1:** Patient characteristics and clinical data

	All patients (n = 219)	Survivors (n = 156)	Non-survivors (n = 63)	P-value
Age (years), mean (± SD)	69.4 ± 13.1	69.1 ± 13.0	70.2 ± 13.5	0.886
Sex, n (%)				0.538
Male	118 (53.9)	82 (52.6)	36 (57.1)	
BMI (kg/m^2^), mean (± SD)	23.0 ± 4.2	22.8 ± 3.9	23.6 ± 4.8	0.091
Charlson comorbidity index, mean (± SD)	5.3 ± 2.6	5.1 ± 2.3	5.9 ± 3.0	0.011
Initial ECOG score, n (%)				0.075
0	52 (23.7)	39 (25.0)	13 (20.6)	
1	59 (26.9)	46 (29.5)	13 (20.6)	
2	47 (21.5)	31 (19.9)	16 (25.4)	
3	38 (17.4)	21 (13.5)	17 (27.0)	
4	23 (10.5)	19 (12.2)	4 (6.3)	
Septic shock, n (%)	103 (47.0)	66 (42.3)	37 (58.7)	0.028
Initial lactate level (mmol/L), mean (± SD)	5.3 ± 4.0	4.5 ± 3.1	7.2 ± 5.0	< 0.001
Initial SOFA score, mean (± SD)	9.5 ± 4.0	8.4 ± 3.1	12.2 ± 4.6	< 0.001
SAPS 3 score, mean (± SD)	73.9 ± 16.4	70.1 ± 14.4	83.4 ± 17.3	0.098
CRRT, n (%)	68 (31.1)	25 (16.0)	43 (68.3)	< 0.001
Steroid use, n (%)	64 (29.2)	42 (26.9)	22 (34.9)	0.239
Initial laboratory results				
Platelet count (10^3^/uL)	170.7 ± 125.6	184.5 ± 130.0	136 ± 107.2	0.299
Creatinine level (mg/dL)	2.1 ± 1.9	1.96 ± 2.0	2.40 ± 1.5	0.796
Total bilirubin level (mg/dL)	3.0 ± 5.1	2.2 ± 2.8	4.9 ± 8.2	< 0.001
CRP level (mg/dL)	14.2 ± 10.3	14.3 ± 9.5	14.0 ± 12.2	0.027
Procalcitonin level (ng/ml)	37.0 ± 67.2	31.7 ± 50.0	50.6 ± 97.9	0.006
BNP level (pg/mL)	699.0 ± 1207.3	458.6 ± 886.0	1312.0 ± 1659.0	< 0.001
Time zero to time of antibiotics administration (min)	184.7 ± 303.0	171.4 ± 238.8	217.5 ± 422.8	0.495
Source control, n (%)	90 (41.1)	73 (46.8)	17 (27.0)	0.007
Source control time (h)	22.1 ± 37.0	22.9 ± 39.4	18.5 ± 24.9	0.489
Number of organ dysfunction, mean (± SD)	1.7 ± 1.3	1.4 ± 1.21	2.3 ± 1.4	0.019

Data are shown as mean ± standard deviation or number (percentage)

*SD* standard deviation, *BMI* body mass index, *ECOG* Eastern Cooperative Oncology Group, *SOFA* Sequential Organ Failure Assessment, *SAPS 3* simplified acute physiology score III, *CRRT* continuous renal replacement therapy, *CRP* C-reactive protein, *BNP* brain natriuretic peptide

In addition, there was no significant difference between the two groups in the duration from time zero to the administration of antibiotics and source control. However, the number of patients who underwent source control through non-surgical treatment such as percutaneous drainage, or surgical treatment was significantly higher in the survivor group than in the non-survivor group (46.8% vs. 27%, *p* = 0.007). The mean number of organ dysfunction was significantly higher in the non-survivor group than in the survivor group (2.3 ± 1.4 vs. 1.4 ± 1.2, *p* = 0.019).

### Microbiological pathogens (or spectrum) and characteristics

Table 2 shows the distribution of the isolated microbiological pathogens expressed by per species percentage of bacteria and fungi from patients with sepsis due to IAI. Of the 219 patients, 157 (71.7%) were identified with a causative pathogen. Among the patients with isolated pathogens, gram-negative bacteria were found in 81.5%, gram-positive bacteria in 32.5%, and fungi in 4.5% of patients. Among the isolated causative pathogens in our study, the most common pathogen was *Escherichia coli* (48.4%), followed by *Klebsiella pneumoniae* (22.9%), *Enterococcus faecium* (8.9%), and *Enterococcus faecalis* (5.7%). Additionally, *Acinetobacter baumannii* (4.5%), *Enterobacter cloacae* (3.8%), *Klebsiella oxytoca* (3.2%), and *Pseudomonas aeruginosa* (3.2%) were other gram-negative bacteria, and *Staphylococcus aureus* (1.8%) and *Corynebacterium striatum* (0.5%) were other gram-positive bacteria. Moreover, *Candida albicans* (3.2%), *Candida glabrata* (2.5%), and *Candida krusei* (0.6%) were the identified fungi.

**Table 2:** Distribution of the microbiological pathogens isolated from cultures in patients with sepsis due to IAI

	No. of patients (total, n = 157)	% Total
Gram-positive	51	32.5
* Enterococcus faecium*	14	8.9
* Enterococcus faecalis*	9	5.7
* Staphylococcus aureus*	4	1.8
* Corynebacterium striatum*	1	0.5
Others	26	11.9
Gram-negative	128	81.5
*Escherichia coli*	76	48.4
*Klebsiella pneumoniae*	36	22.9
*Acinetobacter baumannii*	7	4.5
*Enterobacter cloacae*	6	3.8
*Klebsiella oxytoca*	5	3.2
*Pseudomonas aeruginosa*	5	3.2
Others	24	15.3
Fungus	7	4.5
*Candida albicans*	5	3.2
*Candida glabrata*	4	2.5
*Candida krusei*	1	0.6

Bacteremia occurred in 49.3% of all patients, and did not differ significantly between the survivor and non-survivor groups (48.7% vs. 50.8%, *p* = 0.781). Among the identified pathogens, there were no statistically significant differences in the types of bacteria and fungus between the survivor and the non-survivor groups. Mixed growth was defined as the detection of more than one type of gram-positive, gram-negative, and fungal pathogens. Of the patients with identified causative pathogens, 19.1% had a mixed growth on culture. The proportion of patients with mixed growth was higher in the non-survivor group than in the survivor group, although the difference was not statistically significant (27.5% vs. 16.2%, *p* = 0.304). MDR was defined as the antimicrobial resistance of a microorganism to at least one antibiotic in three or more antimicrobial categories [12]. The overall prevalence of MDR pathogens did not differ significantly between the survivor and non-survivor groups (47.9% vs. 40.0%, *p* = 0.134). Among the MDR pathogens, the most common were *Enterobacteriaceae* (54.2%), followed by *Enterococcus* spp*.* (12.5%), *Acinetobacter* spp. (4.2%), *Staphylococcus aureus* (2.8%), and *Pseudomonas aeruginosa* (2.8%). There was no difference in the mortality rate according to the location of infection (Table 3).

**Table 3:** Microbiological profile on survivors and non-survivors

	All patients (n = 219)	Survivors (n = 156)	Non-survivors (n = 63)	P-value
Identified pathogens, n (%)	157 (71.1)	117 (75.0)	40 (63.5)	0.087
Bacteremia, n (%)	108 (49.3)	76 (48.7)	32 (50.8)	0.781
Type of bacteria, n (%)				
Gram-positive	51 (32.5)	34 (29.1)	17 (42.5)	0.112
Gram-negative	128 (81.5)	95 (81.2)	33 (82.5)	0.322
Fungus, n (%)	7 (4.5)	6 (5.1)	1 (2.5)	0.390
Mixed growth, n (%)	30 (19.1)	19 (16.2)	11 (27.5)	0.304
MDR, n (%)	72 (45.9)	56 (47.9)	16 (40.0)	0.134
* Enterobacteriaceae*	39 (54.2)	30 (53.6)	9 (56.3)	0.387
* Enterococcus* spp.	9 (12.5)	5 (8.9)	4 (25.0)	0.289
* Acinetobacter* spp.	3 (4.2)	2 (3.6)	1 (6.3)	0.860
* Staphylococcus aureus*	2 (2.8)	1 (1.8)	1 (6.3)	0.505
* Pseudomonas aeruginosa*	2 (2.8)	2 (3.6)	0	0.367
Others	21 (29.2)	18 (32.1)	3 (18.8)	0.123
Location of infection, n (%)				
Community-acquired	135 (61.6)	96 (61.5)	39 (61.9)	0.960
Healthcare-acquired	84 (38.4)	60 (38.5)	24 (38.1)	0.960

*MDR* multidrug resistance

### Organ dysfunction

In the organ dysfunction analysis, the most common type of organ dysfunction in patients with sepsis due to IAI was respiratory dysfunction (36.5%), followed by renal (36.1%), coagulation (34.2%), cardiovascular (25.6%), central nervous system (CNS) (19.6%), and liver (18.3%) dysfunctions. Among the dysfunctional organs, the mortality rate associated with each organ dysfunction was the highest in patients with CNS dysfunction, followed by coagulation, renal, respiratory, cardiovascular, and liver dysfunctions (Table 4).

**Table 4:** Organ dysfunction analysis data in sepsis due to IAI

	All patients (n = 219)	Survivors (n = 156)	Non-survivors (n = 63)	P-value
*Organ dysfunction, n (%)*				
Respiratory	80 (36.5)	50 (62.5)	30 (37.5)	0.030
Coagulation	75 (34.2)	42 (56.0)	33 (44.0)	< 0.001
Liver	40 (18.3)	29 (72.5)	11 (27.5)	0.845
Cardiovascular	56 (25.6)	36 (64.3)	20 (35.7)	0.183
CNS	43 (19.6)	24 (55.8)	19 (44.2)	0.013
Renal	79 (36.1)	49 (62.0)	30 (38.0)	0.024

*IAI* intra-abdominal infection, *CNS* central nervous system

Multivariate logistic regression analysis was used to estimate the association between organ dysfunction and 28-day mortality. Among the organ dysfunctions, the 28-day mortality rate was more significantly affected with coagulation (OR = 2.99 [1.63–5.48], *p* < 0.001), CNS (OR = 2.38 [1.19–4.75], *p* = 0.014), renal (OR = 1.99 [1.09–3.61], *p* = 0.024), and respiratory (OR = 1.93 [1.06–3.5], *p* = 0.031) dysfunctions. Furthermore, after adjusting for age, sex, CCI, and lactic acid level, only coagulation dysfunction appeared to affect 28-day mortality significantly (OR = 2.78 [1.47–5.23], *p* = 0.001) (Table 5).

**Table 5:** Multivariable logistic regression analysis for each organ dysfunction in patients with sepsis due to IAI

Variable	Crude		Adjusted	
	OR (95% CI)	P	OR (95% CI)	P-value
Respiratory	1.93 (1.06–3.5)	0.031	1.71 (0.9–3.25)	0.102
Coagulation	2.99 (1.63–5.48)	< 0.001	2.78 (1.47–5.23)	0.001
Liver	0.93 (0.43–1.99)	0.845	0.99 (0.44–2.25)	0.979
Cardiovascular	1.55 (0.81–2.96)	0.185	1.35 (0.67–2.71)	0.398
Central nervous system	2.38 (1.19–4.75)	0.014	1.79 (0.85–3.79)	0.127
Renal	1.99 (1.09–3.61)	0.025	1.45 (0.76–2.76)	0.260

Adjusting was performed according to age, sex, CCI, and lactic acid level

*IAI* intra-abdominal infection, *OR* odds ratio, *CI* confidence interval, *CCI* Charlson Comorbidity Index

On studying the association between the number of organ dysfunctions and the 28-day mortality rate, mortality was found to be 24.7% (n = 20/81, *p* = 0.307) in patients with one organ dysfunction and 18.4% (n = 9/49, *p* = 0.068) in patients with two organ dysfunctions. The 28-day mortality was 55.6% in patients with three organ dysfunctions (OR = 9.06 [2.64–31.15], *p* = 0.001) and 50% in patients with four or more organ dysfunctions (OR = 7.25 (1.85–28.36], *p* = 0.004). In patients with three or more organ dysfunctions, 28-day mortality was more than twice that of single organ dysfunction (Table 6). In time, the mortality rate in patients with more than three organ dysfunctions distinctly increased.

**Table 6:** OR of multiple organ dysfunctions for 28-day mortality in logistic regression analysis

Number of organ dysfunction	n	Mortality	OR (95% CI) Crude	P-value
0	33	4/33 (12.1)	Reference	
1	81	20/81 (24.7)	2.38 (0.74–7.59)	0.144
2	49	9/49 (18.4)	1.63 (0.46–5.81)	0.451
3	36	20/36 (55.6)	9.06 (2.64–31.15)	0.001
≥ 4	20	10/20 (50.0)	7.25 (1.85–28.36)	0.004

*OR* odds ratio, *CI* confidence interval

C-statistics = 0.69, Hosmer–Lemeshow test *p* = 1.000

### Predictive factors for 28-day mortality

CCI, septic shock, initial lactate level, initial SOFA score, SAPS 3 score, CRRT, source control, and the number of dysfunctional organs were associated with 28-day mortality in the univariate analyses. In the multivariate logistic regression analysis, after adjusting for each risk and confounding factor, only the SAPS 3 score (*p* < 0.001) and CRRT (*p* < 0.001) were independently associated with higher 28-day mortality (Table 7).

**Table 7:** Multivariable logistic regression analysis for 28-day mortality in patients with sepsis due to IAI

Variable	Univariable		Multivariable	
	OR (95% CI)	P-value	OR (95% CI)	P-value
Age (years), mean (± SD)	1.01 (0.98–1.03)	0.581		
BMI (kg/m^2^), mean (± SD)	1.04 (0.97–1.12)	0.239		
Charlson comorbidity index, mean (± SD)	1.13 (1.00–1.26)	0.039		
Septic shock, n (%)	1.94 (1.07–3.51)	0.029		
Initial lactate level (mmol/L), mean (± SD)	1.19 (1.09–1.3)	< 0.001		
Initial SOFA score, mean (± SD)	1.24 (1.12–1.36)	< 0.001		
SAPS 3 score, mean (± SD)	1.05 (1.03–1.08)	< 0.001	1.04 (1.02–1.07)	< 0.001
CRRT, n (%)	109.58 (24.89–482.48)	< 0.001	7.71 (3.76–15.82)	< 0.001
Bacteremia, n (%)	1.09 (0.61–1.95)	0.780		
Time zero to time of antibiotic administration (min)	1.00 (1.00–1.01)	0.330		
Source control, n (%)	2.38 (1.26–4.51)	0.008	2.02 (0.95–4.30)	0.069
Source control time (h), mean (± SD)	1.00 (1.00–1.00)	0.660		
Number of organ dysfunction, n (%)	1.66 (1.30–2.13)	< 0.001		
MDR, n (%)	0.61 (0.32–1.17)	0.136		

*IAI* intra-abdominal infection, *OR* odds ratio, *CI* confidence interval, *BMI* body mass index, *SOFA* Sequential Organ Failure Assessment, *SAPS 3* simplified acute physiology score III, *CRRT* continuous renal replacement therapy, *MDR* multidrug resistance

Multiple logistic: C-statistics = 0.83, Hosmer–Lemeshow test *p* = 0.53

## Discussion

Despite the increased incidence of sepsis and septic shock, recent studies have consistently shown a decrease in sepsis mortality over time, owing to advances in medical technology [1, 13]. However, the overall in-hospital mortality rate due to sepsis was still quite high at 29.0% in some recent studies in Korea [10, 14]. Similarly, our study showed that the overall in-hospital mortality rate with sepsis was 28.1%, and the mortality rate for sepsis caused by IAI was 23.3%. In addition, in the ICU, the overall sepsis mortality rate was 35.1%, and the mortality rate of sepsis due to IAI was 28.8%. The finding of higher sepsis mortality rates in the ICU is not surprising because patients treated in the ICU tend to have more severe disease than patients treated in the wards, but more efforts should be made to reduce the high mortality rates.

There have been many studies on tools for predicting the progress and prognosis of the disease, which are still underway [15–17]. One of the most important factors affecting the mortality and clinical outcomes in sepsis is comorbidity, and the CCI can quantify a patient's comorbidity. In this time of aging populations, the CCI of patients is increasing. The CCI is widely used as a tool to predict the mortality rate of patients with sepsis due to IAI and determine their prognosis in advance [14, 18, 19]. In our study, the mean CCI in the non-survivor group was higher than that in the survivor group, with significant differences (5.9 ± 3.0 vs. 5.1 ± 2.3, *p* = 0.011). In addition, CCI (*p* = 0.039, OR = 1.13 [1.00–1.26]) was an independent risk factor for mortality prediction in the univariate logistic regression analysis.

Multicenter research and efforts are being conducted worldwide to improve the clinical outcomes of patients with sepsis. For instance, the Surviving Sepsis Campaign released new guidelines for the treatment of sepsis and a new updated “Hour-1 bundle” for sepsis treatment [20, 21]. Immediate resuscitation, initial early screening, antibiotic treatment, and source control are imperative to improve patient outcomes [14]. In addition, initial lactate levels and initial SOFA scores can be used to predict clinical progress and prognosis of sepsis. A large number of studies have already shown that, the higher the initial lactate level and initial SOFA score, the higher the mortality rate in patients with sepsis [22, 23]. In our study, initial lactate level and SOFA score were also independent risk factors in predicting the mortality rate in patients with sepsis caused by IAI (p < 0.001).

In the treatment of patients with sepsis due to IAI, the usage of antibiotics and source control is very important. Adequate and swift antibiotic administration and coverage from the time of recognition of sepsis are vital [24–27]. Although no significant difference was noted, the non-survivor group showed a tendency of delayed antibiotic administration in our study compared with that in the survivor group (217.5 ± 422.8 min vs. 171.4 ± 238.8 min, *p* = 0.495). Sepsis caused by IAI due to biliary sepsis, intestinal perforation, postoperative leakage, and intra-abdominal abscess can be controlled using open laparotomy, percutaneous transhepatic biliary drainage, and percutaneous catheter drainage insertion. Ultimately, the prognosis of patients with sepsis depends on the source controls, and the faster the source control, the lower is the mortality rate [28, 29]. Our study showed a significantly higher number of survivors in the source control group (46.8% vs. 27.0%, *p* = 0.007).

In several studies on IAI, *E. coli* and *K. pneumoniae* were the most common gram-negative causative pathogens. In gram-positive pathogens, most of them were *E. faecalis* and *E. faecium* [6, 9]. In our study, the same pathogens were also detected and the proportion was similar, as previously mentioned. In several studies, identifying causative pathogens were not significantly correlated to mortality [10, 30, 31]. Likewise, in our study, there was no significant difference in the identification of causative pathogens between the survivor group and the non-survivor group. However, a study by Gupta et al. [32] showed that the mortality rate was higher in patients in whom the causative pathogen was not identified, and that non-identification of the pathogen was an independent predictor of death. Additionally, some studies on IAI have shown that MDR is an independent risk factor for mortality [6, 9, 33]. However, the impact of MDR on mortality was not significant in our study (*p* = 0.136).

Organ dysfunction is a useful prognostic indicator for mortality in patients with sepsis, and the mortality rate increases significantly as the number of organ dysfunctions increases [34–36]. In one study by Umegaki et al. [34], patients with three (23.5%) and four or more organ dysfunctions (38.9%) had over two and four times the ICU mortality rates, respectively, compared with that of single organ dysfunction (8.9%). In addition, the hazard ratios were 1.6, 2.0, and 2.7 in 2-, 3-, and 4 or more organ dysfunctions, respectively, showing an increasing trend as the number of organ dysfunctions increases. In our study, the mortality rate was more than two-fold higher in patients with three organ dysfunctions (55.6%) or four or more organ dysfunctions (50.0%) than that of patients with a single organ dysfunction (24.7%) or two organ dysfunctions (18.4%). The ORs were also 9.06 (*p* = 0.001) and 7.25 (*p* = 0.004) in patients with three and four or more organ dysfunctions, respectively. Similarly, the dysfunction of three or more organs in our study could be considered as an independent risk factor for mortality.

The impact of each organ dysfunction on mortality and the proportion of organ dysfunction occurring in patients with sepsis varies between the studies. In several studies of organ dysfunction in patients with sepsis, respiratory and cardiovascular dysfunction were the most common [13, 34]. However, in our study of patients with sepsis due to IAI, respiratory and renal dysfunction were the most common. In some studies of patients with sepsis, respiratory and cardiovascular dysfunctions had a higher mortality rate than that of patients with other organ dysfunctions [13, 34–36]. However, in our study, the mortality rate was higher in patients with CNS or coagulation dysfunction than in patients with other organ dysfunctions. There are few studies on the impact of each organ dysfunction on mortality in sepsis caused by IAI, so the exact mechanisms by which different organ dysfunctions are associated with death are poorly understood. However, some other studies have shown a higher mortality rate in patients with CNS or coagulation dysfunction than in patients with respiratory or cardiovascular dysfunction [36, 37], as in our study.

The coagulation cascade may be abrogated owing to the aberrant expression of cytokines and tissue factors in response to sepsis with systemic inflammation. Microvascular thrombosis and ischemia are the most important processes in sepsis, causing tissue damage and multiple organ dysfunctions [38, 39]. As the coagulation system is disordered, sepsis-induced coagulopathy occurs, and manifestations of bleeding increase [38–40]. Several studies have shown that coagulopathy in patients with sepsis is associated with high mortality rates [41, 42]. In our study, coagulation dysfunction was closely related to mortality, which may have been due to sepsis-induced coagulopathy or bleeding tendency. However, in this study, data were not collected on the cause of death, so it is unknown whether the deaths were caused by bleeding.

This study has several limitations. First, this was a retrospective study based on medical records conducted in multiple institutions. Each hospital differed in its treatment policy, level, and system, including its facilities. As this study did not only focus on sepsis caused by IAI, the data may also be inadequate. To overcome these limitations, a prospective study of patients with sepsis caused by IAIs may be needed.

## Conclusions

In our study, CCI, septic shock, initial lactate level, initial SOFA score, SAPS 3 score, acute kidney injury with CRRT, source control, and the number of dysfunctional organs were found to be independent risk factors affecting 28-day mortality. Among the organ dysfunctions in sepsis caused by IAI, coagulopathy was found to be an independent risk factor for 28-day mortality. Therefore, more intensive care may be needed because the prognosis could be worse in patients with coagulopathy than in those with other organ dysfunctions due to IAI.

## Data Availability

The datasets generated during and/or analyzed during the current study are available from the corresponding author on reasonable request.

## Abbreviations

IAI: Intra-abdominal infection
ICUs: Intensive care units
KSA: Korean Sepsis Alliance
KDCA: Korea Disease Control and Prevention Agency
SOFA: Sequential Organ Failure Assessment
BMI: Body mass index
CCI: Charlson Comorbidity Index
SAP 3: Simplified acute physiology score III
MDR: Multidrug resistance
ORs: Odds ratios
CRRT: Continuous renal replacement therapy
CNS: Central nervous system
